# Resilience as a predictor of habituation

**DOI:** 10.1007/s00406-023-01658-y

**Published:** 2023-08-01

**Authors:** Christoph Rösner, Oliver Tüscher, Katja Petrowski

**Affiliations:** 1 Medical Psychology & Medical Sociology, University Medical Center of the Johannes Gutenberg, University Mainz, Duesbergweg 6, 55128 Mainz, Germany; 2 Leibniz Institute for Resilience Research (LIR) gGmbH Mainz, Wallstr. 7, 55122 Mainz, Germany; 3 Department of Psychiatry and Psychotherapy, University Medical Center of the Johannes Gutenberg, University Mainz, Untere Zahlbacher Str. 8, 55131 Mainz, Germany; 4 Institute of Molecular Biology gGmbH Mainz, Ackermannweg 4, 55128 Mainz, Germany

**Keywords:** Cortisol, Resilience, Trier Social Stress Test (TSST), Repeated stress, Habituation, Hypothalamus–pituitary–adrenal axis reactivity

## Abstract

Habituation refers to the physiological adaptation to recurrent stressors, which can be measured by cortisol levels, and is considered a central mechanism in reducing allostatic load. Resilience, a potential factor influencing stress reduction, is the focus of this study. Specifically, the study aims to investigate the impact of resilience, as assessed by the Brief Resilience Scale (BRS), on habituation. The Trier Social Stress Test (TSST) was used as the recurrent stressor, and it was administered to each of the 56 subjects at 4 consecutive measurements. To assess habituation, various physiological parameters including the area under the curve with respect to the ground (AUCg) and with respect to the increase (AUCi), cortisol peak, slope from baseline to peak, and recovery were calculated. Mixed linear models were employed to examine the differences in the influence of resilience on habituation across the different time points. The findings indicate that the influence of resilience significantly varies from the first to the fourth measurement time point for AUCg (*p* = .048), while no significant differences were observed for the other cortisol parameters. The effects plot suggests that individuals with higher levels of resilience exhibit lower AUCg values throughout the measurements. These findings provide initial evidence supporting resilience as a predictor of cortisol habituation. However, future studies should also consider dynamic resilience models, utilizing longitudinally assessed resilience as a predictor for habituation, to explore whether resilience acts as a determinant of habituation or if habituation itself constitutes a resilience mechanism.

## Introduction

Stress is a ubiquitous experience in everyday life. While some stressors occur routinely and may not be individually perceived as major stressors, such as waiting for a train or engaging in an argument with a family member [1], there are instances where stress can have profound and wide-ranging consequences. For instance, an increasing burden of work-related stress has been found to contribute to over 15 days of sick leave per year per person in Germany [2]. To maintain mental and physical health, it is crucial to adapt to these stressors in a manner that minimizes their impact based on an individual's own assessment.

The body's capacity to achieve stability in the face of stress through physiological adjustments is known as allostasis [3]. Allostasis involves the integration of various physiological systems, including the sympathetic and parasympathetic systems, the immune system, and the hypothalamic–pituitary–adrenal (HPA) axis [4]. These systems are activated to provide the necessary resources for coping with stressors. The concept of allostatic load refers to the physiological effort expended in response to stressors [5]. The goal is to maintain a balance between the physiological resources needed to cope with the stressor and the available physiological resources. However, when the demands imposed by the stressor exceed an individual's physiological resources, an imbalance called allostatic overload occurs [6]. This allostatic overload can result in long-term changes, such as suppressed neurogenesis or impaired stress recovery [7]. Consequently, a prolonged and inadequate response can trigger an overreaction of other physiological mechanisms [8]. Both psychological and physical symptoms, as well as specific diseases, can arise as a consequence of allostatic overload, including an increased risk of coronary heart disease and psychological disorders [9–11]. Thus, the identification and prevention of allostatic overload are crucial in addressing stress-related disorders.

The identification of biomarkers for allostatic overload can provide valuable insights into the activation of the aforementioned physiological systems [12]. Among the key components of the 'stress system,' the hypothalamic–pituitary–adrenal (HPA) axis plays a prominent role. In brief, the HPA axis operates as follows: the hypothalamus stimulates the production of adrenocorticotropin hormone by releasing corticotropin-releasing hormone and arginine vasopressin, which, in turn, stimulates cortisol production in the adrenal cortex [13]. While cortisol release has various beneficial effects on the stress response, such as immunosuppression and energy mobilization, it is commonly employed as a biomarker due to its well-documented role as a mediator of the stress response [13–15].

To assess excessive demands on an individual's physiological resources or maladaptation, the dynamic response of cortisol levels to repeated stressors can be examined over time [7]. Of particular interest is the cortisol profile, which can exhibit diverse physiological stress responses, including a decrease in cortisol levels over time after repeated exposure to the same stressor, known as habituation, which has been associated with a reduction in the stress response [16, 17]. If habituation is indeed related to allostatic load, it suggests that stressors to which habituation has occurred impose a lower or more manageable burden over time [8, 17]. While habituation has been studied in relation to various physiological systems, it appears to be primarily observed in the context of the HPA axis [18]. Interestingly, habituation of the HPA axis appears to be accompanied by sensitization of the peripheral inflammatory system, indicating both activation and deactivation of physiological systems in reducing allostatic load [19]. However, the type of stressor seems to play a crucial role in cortisol habituation, with physical stressors (e.g., skydiving) more likely to exhibit habituation compared to social stressors (e.g., a competition among ballroom dancers) [20]. Additionally, other factors influencing cortisol habituation to a stressor need to be considered, such as helplessness [21], anxiety [22], and trait reappraisal [23]. Consequently, resilience, as theorized by Bennett et al. [20], may serve as another determinant of cortisol habituation.

Resilience, in the psychological sense, has recently been defined as the ability to maintain mental health or recover despite significant adversity [24–27]. Given that adversity can also be viewed as acute or chronic stress, resilience is inherently linked to physiological systems, including the HPA axis, and thus to the theory of allostatic load [26–28]. Previous studies have already demonstrated significant relationships between resilience, as measured by various resilience questionnaires, and measures of cortisol, such as urinary cortisol [29], hair cortisol concentrations [30], and salivary cortisol [31, 32].

However, in the studies utilizing salivary cortisol [31, 32], a single stress induction was employed, primarily capturing the acute stress response. To further investigate the relationship between resilience and cortisol response, particularly in the context of habituation, it was essential to examine the cortisol profile following repeated stress inductions. The aim was to explore the association between resilience and the cortisol trajectory during stress habituation. Therefore, this study aimed to test the hypothesis that resilience would also predict the cortisol course of stress habituation measured after repeated stress induction. By doing so, this study aimed to expand upon previous findings that focused on the influence of resilience on acute stress processing, by investigating its impact on the processing of chronic stress. Additionally, more comprehensive constructs of cortisol reactivity, such as "total hormonal output" and "sensitivity of the system" established by Pruessner, Kirschbaum, Meinlschmid, and Hellhammer [33], were employed to explore how resilience influences habituation.

## Materials and methods

### Participants

The study protocol was approved by the Ethics Committee of the State Medical Association of Rhineland Palatinate (Landesärztekammer Rheinland-Pfalz), Germany (2019–14433) and in accordance with the Declaration of Helsinki (1964). All subjects received prior information about the procedure and provided written informed consent prior to participation.

The sample (n = 56) included young, healthy male adults (mean age 24.88, SD 4.16) from Germany. As inter-individual variabilities (e.g., health status, smoking, age, BMI, menstrual cycle phase) are known to affect HPA axis responses of humans [33–35], only young males were chosen to exclude gender and age effects [36]. A brief description of the sociodemographic characteristics of this sample is provided in Table 1. The participants were recruited via posters and online channels and received a compensation of 75 euros. They underwent a preliminary telephone screening for health assessment focusing on the predefined exclusion criteria of this study (such as chronic illness, psychiatric disorders, dependency on alcohol or drugs, smoking ≥9 cigarettes daily, body mass index [BMI] not between 18 and 27 kg/m^2^, age range not between 18 and 30 years, medication [e.g., anti-depressants, beta-blockers], familiarity with stress tests). For sample size estimation, a statistical power analysis, i.e., G*Power version 3.1.9.7 [37], was applied. The estimated total sample size resulted in at least 36 participants for a repeated measures ANOVA with an effect size of 0.25 and an α error of 0.05.

**Table 1:** Sample characteristics

Variable	*n*	M	SD
Age (in years)	56	24.88	4.16
BMI (kg/m^2^)	56	23.24	2.23
Doing sports	40		
Sleep problems	13		
In a relationship	31		
Professional degree			
Student or apprentice	19		
Professional school diploma	9		
Bachelor/Master/PhD	*19*		
Without vocational qualification	*8*		
Employment			
Employed	*11*		
Mini-job	*28*		
Unemployed	*8*		

Bold represents that it is statistically significant

### Procedure (stress test)

**Fig. 1:**
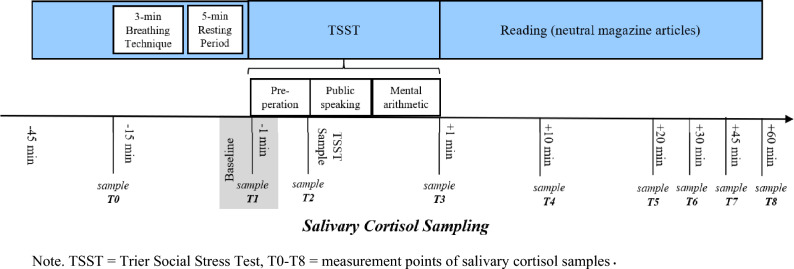
Study design and cortisol measurement points. *TSST* Trier Social Stress Test, *T0–T8* measurement points of salivary cortisol samples

The participants were scheduled individually using a within-subject experimental study design. Salivary cortisol samples were collected throughout the testing period on each day. A predefined cortisol sampling timeframe between 1:00 and 5:00 p.m. was chosen to reduce circadian rhythmicity effects on cortisol measurements. The participants were instructed via e-mail to refrain from alcohol consumption and any strenuous physical activity/exercise 24 h prior to the laboratory days and from eating or drinking 1 h before their laboratory visits. Upon arrival, the participants sat down for 45 min as a baseline before they were introduced to the Trier Social Stress Test (TSST) by Kirschbaum, Pirke, and Hellhammer [38]. The psychosocial stress protocol includes a preparation time followed by a 5-min job interview situation and a subsequent 5-min mental arithmetic task in front of a two-person panel (see [39, 40], for details). 30 min after arrival, the first salivary cortisol sample was taken (-15 min) followed by the baseline sample (-1 min) directly before the TSST and one sample during the TSST (0 min). Saliva samples were repeatedly obtained 1, 10, 20, 30, 45, and 60 min after the TSST. Testing took on average 2 h to complete on each day of laboratory assessment (see overview in Fig. 1). To avoid possible effects of the participants’ presenting a speech by heart, getting used to the panel, or memorizing the arithmetic task, the setting of the stress test was altered minimally (for details see [41]). The job description was modified each time, at least one panel member was exchanged, and the arithmetic task with the initial numbers was changed using a different starting number and subtrahend. Each participant underwent the TSST four times (t1–t4) to induce repeated stress. The second TSST measurement occurred 1 week after the first, the third measurement after 7 weeks, and the fourth measurement after 8 weeks. The questionnaires were filled in electronically using SoSci Survey version 3.2.06.

### Saliva collection and cortisol assessment

Saliva collection was performed with Salivette® swabs (Sarstedt, Nümbrecht, Germany) as a rapid and hygienic collection method. Saliva samples were frozen and stored at -20 degrees Celsius until analysis. After thawing, the Salivettes were centrifuged at 3000 rpm for 5 min, which resulted in a clear supernatant of low viscosity. Salivary concentrations were measured using a commercially available chemiluminescence immunoassay with high sensitivity (IBL International, Hamburg, Germany). The intra- and inter-assay coefficients for cortisol were below 7% and 9%, respectively.

### Resilience

Resilience was assessed at baseline using the German version [42] of the Brief Resilience Scale (BRS) [43]. Unlike other questionnaires, the BRS focuses specifically on resilience as an outcome, aligning with the current definition in the field (as described earlier, e.g., [25–27]). It aims to measure the individual's ability to recover from stress in the face of significant adversity, including chronic stressors or adverse life events. The BRS consists of six items that are rated on a five-point Likert scale (1 = strongly disagree, 2 = disagree, 3 = neutral, 4 = agree, 5 = strongly agree). The German translation of the BRS was developed in 2018 and has demonstrated good reliability in two different samples (N1 = 1128, N2 = 1481) with a Cronbach's alpha coefficient of 0.85 [42]. Factor analysis revealed a single-factor solution for the BRS. Although the BRS shows a low to moderate correlation with optimism and self-efficacy, confirmatory factor analysis confirms that it represents an independent construct, as each construct yields a distinct factor [44].

### Statistical analysis

For the statistical analysis, each cortisol value was transformed by adding one and then logarithmized. Outliers were identified as values more than three standard deviations above or below the mean cortisol values at each time point. These outliers were corrected to three standard deviations. The statistical analysis consisted of two steps. In the first step, paired sample *t *tests were conducted to assess whether the TSST elicited a stress response on all 4 test days.

For the second step, two mathematical parameters, namely "sensitivity of the system" (AUCi) and "total hormonal output" (AUCg), were calculated for each TSST using the method described by Pruessner, Kirschbaum, Meinlschmid, and Hellhammer [33]. In these calculations, the cortisol curve was divided into individual sections, with each section defined by two consecutive cortisol measurements (e.g., section one comprised measurements 1 and 2). The individual sections represented the changes in cortisol values over time. AUCg encompassed the entire area under the curve, while AUCi only considered the change in the sections above the baseline measurement, obtained by subtracting the first cortisol value from all calculated sections. Additionally, the parameters cortisol peak, slope from baseline to peak (SBP), and recovery were calculated for each TSST [45]. SBP was determined as the difference between the first cortisol measurement and the cortisol peak. Recovery was calculated as the difference between the peak and the last cortisol reading, providing a measure of the recovery phase.

In the second step, the effect of resilience on the cortisol parameters was analyzed using a mixed models with the interaction of time and resilience as a fixed effect. The intercept was treated as a random effect to account for intra-individual changes. The analysis was performed with the statistical program jamovi [46]. This analysis aimed to examine whether the effects of resilience on cortisol parameters differed across the four measurement time points. Additionally, an omnibus test on the cortisol parameters was conducted at baseline to assess overall differences in the effect of interest across the time points. The goodness-of-fit measure R2 was reported for these models.

## Results

With regard to the BRS itself, an average of *M* = 3.70 (*SD* = 0.66) was obtained, which, compared to the German norm sample (whole population mean scores between 3.5 and 3.6 around the 50^th^ percentile, stratified by age (18–29) and gender between 3.7 and 3.8 (male)), shows that our sample was on average regarding resilience as measured by the German BRS [44, see Electronic Supplementary Materials ESM 9].

**Fig. 2:**
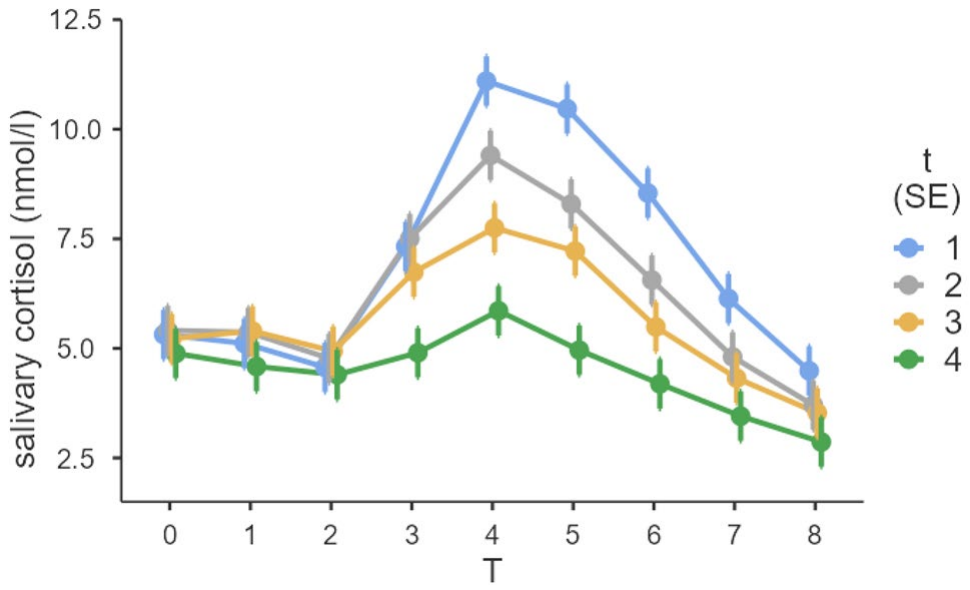
Salivary cortisol responses to four TSST exposures. t2 was 1 week, t3 7 weeks, and t4 8 weeks after t1

In the first step, it was checked whether the TSST was able to elicit a stress response on the 4 test days. A significant difference between the first cortisol measurement and the cortisol peak was found for all four measurement days: TSST day one *t*(55) = − 12.18, *p* < 0.001, TSST day two *t*(55) =  − 8.46, *p* < 0.001, TSST day three *t(*55) =  − 7.67, *p* < 0.001, TSST day four *t*(55) =  − 6.99, *p* < 0.001. The stress response can also be identified by the slope of cortisol levels on all 4 test days from T2 (immediately before TSST) to T4 (10 min after TSST) (Fig. 2). After the peak at T4, cortisol levels decreased again in the recovery phase.

In the second step, mixed models were calculated on cortisol parameters, respectively. The results for the fixed effect parameter measurement points in time (t) interacting with resilience (BRS) are shown in Table 2. Here, intra-individual differences regarding the time points were always relevant with respect to t1. First, the results of the omnibus tests have to be checked, giving information about the occurrence of at least one significant interaction effect between BRS and t [47]. The procedure for linear models implies that further elaboration of the results of the statistical analysis is not warranted if the omnibus does not show significant results. The omnibus test found no significant effect for AUCi (*χ*^2^(3, *N* = 56) = 2.66, *p* = 0.447), peak (*χ*^2^(3, *N* = 56) = 7.56, *p* = 0.056); SBP (*χ*^2^(3, *N* = 56) = 0.91, *p* = 0.823), and recovery (*χ*^2^(3, *N* = 56) = 2.86, *p* = 0.415).

**Table 2:** Mixed model results of the fixed effect parameter of interaction between resilience (BRS) and the time points t2 to t1 (2-1), t3 to t1 (3-1), and t4 to t1 (4-1) for area under the curve with respect to ground (AUCg), respect to increase (AUCi), cortisol peak, slope from baseline to peak (SBP), and recovery (peak to the last cortisol measure T8) as measure of hormonal output

Interaction	β	df	t	p
AUCg				
BRS * 2–1	5.98	160.1	0.801	0.422
BRS * 3–1	− 9.30	160.1	− 1.245	0.212
BRS * 4–1	− 15.05	160.7	− 1.973	**0.048**
AUCi				
BRS * 2–1	11.25	160.0	1.299	0.196
BRS * 3–1	0.68	160.0	0.078	0.938
BRS * 4–1	3.38	161.3	0.382	0.703
Peak				
BRS * 2–1	0.02	162.0	0.164	0.870
BRS * 3–1	− 0.12	162.0	− 1.180	0.240
BRS * 4–1	− 0.23	162.0	− 2.237	**0.027**
SBP				
BRS * 2–1	0.08	162.0	0.70	0.488
BRS * 3–1	− 0.01	162.0	− 0.10	0.918
BRS * 4–1	− 0.01	162.0	− 0.13	0.900
Recovery				
BRS * 2–1	− 0.05	162.0	− 0.663	0.508
BRS * 3–1	− 0.04	162.0	− 0.605	0.546
BRS * 4–1	− 0.12	162.0	− 1.665	0.098

Bold represents that it is statistically significant

**Fig. 3:**
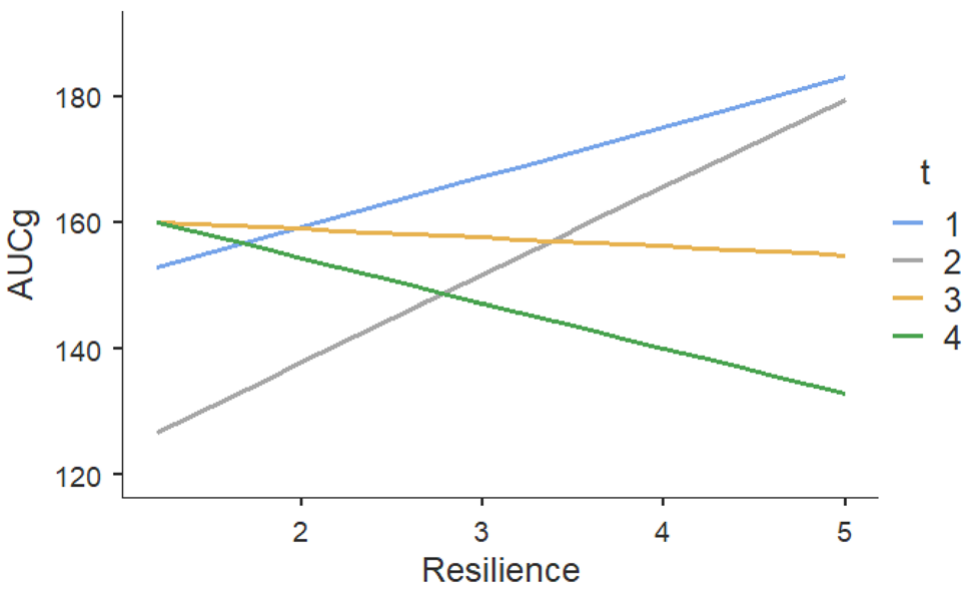
Effects plot of the mixed model with the influence of resilience (BRS) on AUCg across four test points in time (t1-t4)

**Fig. 4:**
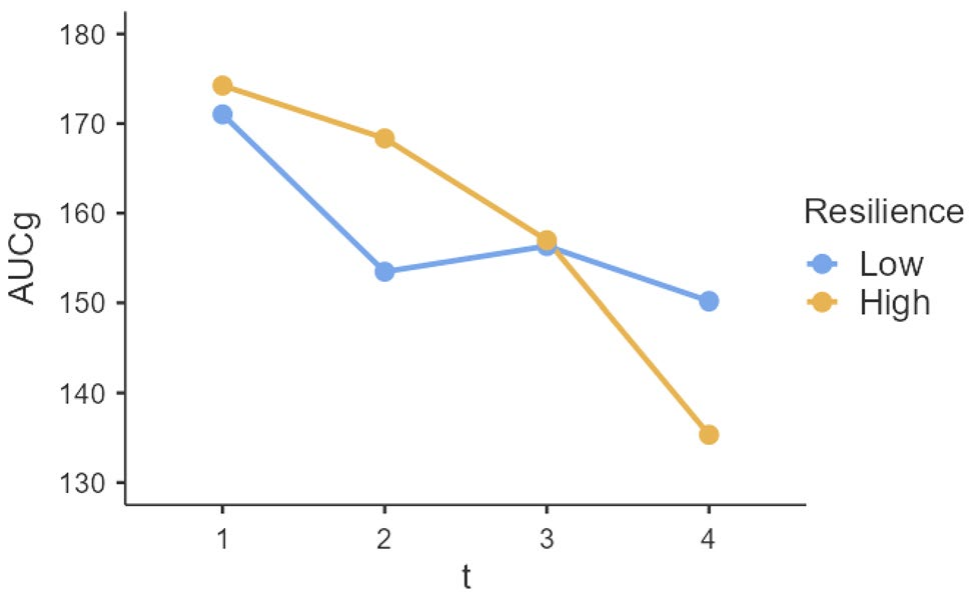
Differences between participants being high and low in resilience for AUCg

For AUCg, the omnibus test indicated that there is at least one significant difference for the influence of resilience at the different time points (*χ*^2^(3, *N* = 56) = 9.22, *p* = 0.027). The model of AUCg yields an *R*^*2*^ = 0.08, which represents a relatively small amount of variance explained for the dependent variable by the independent variables. For t2 (*t*(160.1) = 0.801, *p* = 0.422) and t3 (*t*(160.1) = -1.245, *p* = 0.212), no significant difference could be found with respect to t1 regarding the effect of resilience on AUCg. Only for t4, the difference to time t1 is significant (*t*(160.7) =  − 1.973, *p* = 0.048). This difference between the influence of resilience during t1 and t4 is shown by Fig. 3. Through a post hoc linear regression, it can be seen that as resilience increases during t1, AUCg increases by *β* = 7.91, but at time 4, as resilience increases, AUCg decreases by *β* = -7.14. Also, for t3 AUCg decreases with increasing resilience but not significantly, only by *β* = − 1.38. To make further statements about comparing low- and high-resilient individuals, the metric variable BRS was dichotomized. In Fig. 4, the difference between the groups becomes apparent that high-resilient individuals start with a higher AUCg value than low-resilient individuals. Still, the value continuously decreases in the course, so the value at t4 is even lower.

From a conservative perspective, it is generally not advisable to further examine results after a non-significant omnibus test [47]. However, it is worth noting that in some cases, there may still be significant findings even when the omnibus test does not show significance [48]. In this study, upon further inspection of the post hoc tests, a significant difference was found in peak cortisol levels between t4 and t1 in relation to resilience (*t*(162.0) = − 2.237, *p* = 0.027) (Table 2). Nevertheless, it is important to exercise caution with this result, as disregarding the omnibus test could potentially increase the likelihood of a Type 1 error. Therefore, the significance of this result should be interpreted as a tendency rather than fully conclusive.

## Discussion

Habituation plays a crucial role as a physiological mechanism in reducing allostatic load and is considered a significant factor in the prevention of mental illness [3]. While previous studies have explored the association between cortisol response and resilience, these investigations primarily focused on a single stress induction [30, 31]. Consequently, habituation was investigated to disentangle the relationship between resilience and the course of the cortisol response after multiple presentations of a stressor.

Within the present study, it was observed that resilience played a role in explaining the changes observed in cortisol levels over time during repeated exposure to a stressor. Specifically, resilient individuals showed a significant reduction in total hormonal output (AUCg) from t1 to t4, whereas this reduction was not observed in non-resilient individuals (Table 2). The decreased total hormonal output (AUCg) in resilient individuals may be indicative of their ability to adapt and regulate disturbances, resulting in lower cortisol levels after adaptation [20]. Moreover, a post hoc linear regression analysis revealed a negative relationship between the level of resilience and total hormonal output (AUCg) at the end of the habituation process. This finding supports the hypothesis that resilience and stress-related cortisol habituation are interconnected, suggesting that habituation could serve as a physiological marker or mechanism of resilience [20]. Furthermore, highly resilient individuals exhibited higher cortisol responses at t1 and t2 (see Fig. 4), indicating a more dynamic cortisol response in these individuals in terms of total hormonal output (AUCg) compared to less resilient individuals who displayed relatively "blunted" cortisol reactivity during habituation. However, no significant differences were found in the influence of resilience on the sensitivity of the system (AUCi), slope from baseline to peak (SBP), and recovery over time. For cortisol peak, there appears to be a tendency for varying influences of resilience over time.

To the authors' knowledge, this study is the first to examine the influence of resilience on cortisol dynamics during repeated exposure to social stressors. However, a study conducted by Lü, Wang, and You [49] explored the relationship between resilience and cardiovascular recovery (systolic and diastolic blood pressure) as a marker of habituation to a one-time repeated stressor. Their findings demonstrated that highly resilient individuals exhibited greater reductions in systolic and diastolic blood pressure compared to less resilient individuals. Therefore, resilience might impact both cortisol habituation, as measured in this study, and habituation in terms of cardiovascular recovery. Furthermore, resilience has been shown to affect cortisol reactivity in response to a single stress induction, with more resilient individuals displaying lower cortisol reactivity in both short-term salivary and long-term hair cortisol responses [30–32]. However, based on the available data, it remains unclear whether resilience leads to improved cortisol habituation or if individuals with more efficient HPA axis habituation are more likely to be resilient. This question could be addressed by longitudinal studies that assess resilience concurrently with repeated stress-induced cortisol habituation [26–28, 50].

Resilience appears to play a crucial role in reducing the physiological burden associated with coping with stressors, thereby preventing an excessive or inadequate stress response [8]. By reducing the physiological demands required to handle stressors or maintain allostatic load [5], resilience helps to maintain a state of balance between the available physiological resources and the demands placed upon them. As a result, resilience acts as a protective factor against allostatic overload [6]. This suggests that resilience may serve as a key factor in the prevention of various diseases that are associated with allostatic overload, including stress-related mental disorders.

This study has several strengths, including its high level of standardization and the repeated administration of four standardized psychosocial stress inductions. These rigorous procedures enhance the reliability and validity of the study's findings. The results contribute to the existing literature by expanding our understanding of how resilience influences stress reactivity and the habituation of the HPA axis, which is a critical physiological process in reducing allostatic load. However, there are limitations to consider. One limitation is that we only measured a single parameter of allostatic load, specifically the HPA axis. It would be beneficial to include additional parameters such as heart rate to provide a more comprehensive assessment. Another limitation is the cross-sectional measurement of resilience using the Brief Resilience Scale (BRS), which assesses resilience as a trait rather than a state. This may have restricted the ability of the BRS to predict changes in other cortisol parameters, as it primarily captured the trait-like aspect of resilience. Future studies may benefit from incorporating more comprehensive and dynamic measures of resilience to better capture its influence on various aspects of stress reactivity.

In future research, it is recommended to adopt a longitudinal research design when measuring resilience. Rather than assessing resilience only once, multiple measurements over time would allow for a more comprehensive understanding of the dynamic nature of resilience and its impact on health outcomes [50, 51]. By incorporating repeated measurements, researchers can examine how individual health changes from pre-measurement to post-measurement, shedding light on the underlying processes and mechanisms involved. A valuable parameter in studying dynamic resilience is the Stressor Reactivity Score, which has been recently introduced. This score captures the fluctuating nature of resilience and provides insights into how individuals respond to and recover from stressors [28]. Incorporating this dynamic resilience concept into longitudinal studies would enable researchers to attribute differences in health outcomes to specific traits, capabilities, and resilience mechanisms measured over time [26, 27]. Therefore, it is crucial for future studies to embrace the concept of dynamic resilience and explore its potential in elucidating the various aspects and determinants of habituation. By adopting a longitudinal approach, researchers can uncover new insights into resilience and its implications for stress reactivity, habituation, and overall well-being.

In summary, this study examined the impact of resilience on habituation to a recurrent stressor using the TSST paradigm. The findings provided initial evidence that resilience may play a role in influencing habituation, specifically in terms of total hormonal output. This study highlights the importance of investigating the dynamic relationship between resilience and habituation in future research. Exploring the reciprocal influence of the dynamic resilience concept on habituation, or vice versa, would provide valuable insights into the interplay between these factors. Further investigations are needed to deepen our understanding of how resilience and habituation interact and contribute to stress response and adaptation.

## Data Availability

The data are not publicly available due to confidentiality.
